# Speciation and antifungal susceptibility of *Candida* isolates from diabetic foot ulcer patients in a tertiary hospital in Kenya

**DOI:** 10.11604/pamj.2022.41.34.30815

**Published:** 2022-01-13

**Authors:** Victor Moses Musyoki, Winnie Mutai, Nancy Ngugi, Fredrick Otieno, Moses Muia Masika

**Affiliations:** 1 Department of Medical Microbiology, School of Medicine, College of Health Sciences, University of Nairobi, Nairobi, Kenya,; 2 Department of Medicine, Kenyatta National Hospital, Nairobi, Kenya,; 3 Department of Clinical Medicine and Therapeutics, School of Medicine, College of Health Sciences, University of Nairobi, Nairobi, Kenya,; 4 KAVI-Institute of Clinical Research, College of Health Sciences, University of Nairobi, Nairobi, Kenya

**Keywords:** Diabetic foot ulcer, *Candida* species, antifungal resistance, susceptibility, Fungi

## Abstract

**Introduction:**

diabetic foot ulcer is the leading cause of hospital admissions, lower limb amputation and death among diabetic patients. Little information is available on fungal isolation in diabetic foot ulcer patients, especially in sub-Saharan Africa. This study aimed to describe Candida species infecting diabetic foot ulcers in patients receiving clinical care at Kenyatta National Hospital and assess their antifungal susceptibility profile.

**Methods:**

this was a cross-sectional study carried out at Kenyatta National Hospital among adult diabetic foot ulcer patients over a three-month period. Species identification of Candida was performed using VITEK - 2 System and further confirmed by Matrix-Assisted Laser Desorption Ionization-Time of Flight Mass Spectrometry. Antifungal susceptibility testing was determined using VITEK-2 System. Data were analysed using WHONET and SPSS.

**Results:**

among the 152 study patients recruited, 98% (n=149) had type 2 diabetes. Sixty one percent of the participants were male. The mean age of the study participants was 50.7 years (SD 12.9). A total of 36 Candida species were isolated, of which 75% (n=27) were Candida albicans. Candida lusitaniae (8%, n=3) and C. dubliniensis (5%, n=2) were the predominant non-albicans Candida species. The overall prevalence of diabetic foot ulcer candidiasis was 20% (n=31). C. albicans isolates (26%) were resistant to caspofungin, fluconazole, micafungin, and voriconazole but highly susceptible to amphotericin B and flucytosine (81-96%). Non-albicans Candida species isolated were susceptible (90-100%) to a majority of the antifungal agents tested.

**Conclusion:**

Candida albicans was the predominant species isolated and showed low resistance rates to the commonly administered antifungal agents. There is need to include fungal diagnosis in the investigation of diabetic foot ulcer infection.

## Introduction

Diabetes mellitus is a metabolic disorder characterized by chronic hyperglycemia [1-3] and severe complications [3]. Globally, 1.6 million deaths occur every year due to diabetes and diabetes-related complications, with more than 80% occurring in low and middle-income countries [4,5].

Diabetic foot ulcer (DFU) is the most common complication and leading cause of hospitalization and non-traumatic lower limb amputations. It´s estimated that 10-15% of diabetic patients will develop DFU during their diabetic life [5-7]. According to a recent meta-analysis, the overall prevalence of DFUs is about 13%, while in Kenya, Nyamu *et al*. reported a prevalence of 4.6%, slightly lower than what was previously reported in Tanzania (7.3%) and Egypt (6.2%) [8,9]. The foot ulcer is a significant predisposing factor for microbial infections [10] and early diagnosis and treatment of microbial agents with appropriate antimicrobial therapy is essential. In most instances, the infected wounds are polymicrobial in nature, and information on infecting bacterial agents is available from studies done in both developed and developing countries [11]. Although culture dependent techniques have been widely utilized in isolating infecting microorganisms, major focus has been on *Staphylococcus aureus* and gas gangrene associated *Clostridium perfringens* creating isolation biasness [10,12].

Lack of guidelines on diagnosis and treatment of DFU for microorganisms including fungi, especially in sub-Saharan Africa has contributed largely to the paucity of data on fungal agents infecting DFU. Fungal DFU infection poses a major health concern with antifungal resistance complicating management of these infections and significantly increasing incidence rates of lower limb amputation despite the proper surgical and antimicrobial therapy [13,14]. *Candida* species is the principal fungal pathogen with non-*albicans Candida* (NAC) species emerging as important pathogens isolated from DFU [15,16]. Multidrug resistant *Candida albicans* and pandrug resistant *C. auris* cases have been reported in Asia, South Africa and United States and have led to increased length of hospital stay and healthcare costs [17]. Antimicrobial resistance remains a global health problem and a threat to management of diabetic foot infections [18,19]. Identification of *Candida* species and the primary antifungal drugs to use for treatment in diabetic foot infection is essential in reducing the cost of managing diabetic foot ulcers, amputation and monitoring of antifungal drug resistance.

This study was conducted to determine the species and antifungal susceptibility pattern of *Candida* isolated from diabetic foot ulcers in patients receiving clinical services at Kenyatta National Hospital in Nairobi, Kenya.

## Methods

**Study design and subjects:** this was a cross sectional study carried out in Kenyatta National Hospital (KNH), a tertiary teaching and referral hospital in Nairobi, Kenya. Using Cochran sample size calculation for finite population correction and KNH DFU records (180) for three months, we consecutively recruited adult diabetic patients, both outpatient and inpatient with any type of diabetes presenting with acute and chronic non-healing foot ulcers. Chronic foot ulcers were defined as wounds that did not heal within 3 (three) months. Patients who were on immunosuppressive drugs/state, systemic or topical antimicrobial agents for more than one week at the time of enrolment were excluded.

**Data and sample collection:** clinical and socio-demographic data was collected using a structured questionnaire after obtaining written informed consent. A total of 152 non-repetitive deep tissue samples were collected over a three-month period from each patient using sterile cotton swabs (levine technique) after debriding and cleansing the wound with normal saline (NaCl, 0.9%). The swabs were taken to University of Nairobi Microbiology Laboratory within 2 hours for analysis.

**Microbiological methods:** two smears from each sample were prepared and examined microscopically in 10% potassium hydroxide (KOH) and gram staining. The specimens were inoculated on Sabouraud Dextrose Agar (SDA) media supplemented with chloramphenicol and gentamicin and incubated under aerobic conditions at 37°C for 24-48 hours. Growth on SDA was evaluated for colonial morphology and the yeast identified by conventional methods including germ tube test, microscopic morphology on KOH and gram staining. Further identification including speciation was done using VITEK-2 System (YST card) and Matrix-Assisted Laser Desorption Ionization-Time of Flight Mass Spectrometry (MALDI-TOF MS). Antifungal susceptibility testing of *Candida* isolates was done using the VITEK® 2 System (AST-YS08) (BioMérieux, France) and analyzed according to the 2017 Clinical and Laboratory Standards Institute guideline (CLSI M60). The panel of antifungal agents tested included amphotericin B, caspofungin, fluconazole, flucytosine, micafungin and voriconazole. *C. albicans* ATCC 10231 and *C. parapsilosis* ATCC 22019 were used as controls during the laboratory procedure.

**Statistical analysis:** identification and antifungal susceptibility data were retrieved from the VITEK® 2 System and imported to WHONET (version 5.6) through BACLINK software. Analysis was done using WHONET and IBM SPSS Statistics version 21. Frequency distribution and proportions was done for categorical variables such as gender, type of diabetes and measures of central tendency for numerical variables such as age. Chi-square was done in bivariate analysis to assess any association between categorical variables. Confidence intervals were calculated using the Agresti-Coull interval as recommended in the CLSI M60 document which details analysis and presentation of cumulative antimicrobial susceptibility test data.

**Ethics statement:** this study was approved by the Kenyatta National Hospital-University of Nairobi Ethics and Research Committee (P290/04/2019). Permission to conduct the study in Kenyatta National Hospital was granted by the Head of Department, Medicine and the Head of Diabetes and Endocrine Clinic, Kenyatta National Hospital. Patients were enrolled in the study after written informed consent was obtained. Data was collected using anonymous questionnaires and no personal identifiers were analyzed.

## Results

**Patient demographics:** a total of 152 patients met the inclusion criteria with nearly all patients presenting with type 2 diabetes (98%, n=149). Majority of these patients were male (61%, n=93) and urban dwellers (74%, n=113). The mean age of the study participants was 50.7 years (SD, 12.9). The median duration of diabetes and diabetic foot ulcer was 11 years (IQR 5.25-11.0) and 2 months (IQR 1.0-3.0) respectively. Median random blood sugar level was 7.10 mmol/L (IQR 5.93-8.19). Approximately 20% (n=30) of the patients were on antibiotic treatment mainly metronidazole (80%, n=24), as prophylaxis while none was on antifungal drug (Table 1).

**Table 1:** socio-demographic and clinical characteristics of diabetic foot ulcer patients receiving clinical care at Kenyatta National Hospital (N=152)

	n (%)
**Age group**	
<40	35 (23%)
40-50	47 (31%)
50-60	36 (24%)
>60	34 (22%)
**Gender**	
Male	93 (61%)
Female	59 (39%)
**Residence**	
Urban	113 (74%)
Rural	39 (26%)
**Type of diabetes**	
Type 1	3 (2%)
Type 2	149 (98%)
**Duration of diabetes (years)**	
<5	32 (21%)
5-10	34 (22%)
10-15	42 (28%)
>15	44 (29%)
**Duration of diabetic foot ulcer (months)**	
<3	123 (80%)
3-5	19 (13%)
>5	10 (7%)
**Wagner ulcer grade**	
I	40 (26%)
II	93 (61%)
III	13 (9%)
IV	6 (4%)
**Episode of DFU**	
I	58 (38%)
II	88 (58%)
III	6 (4%)
**Medication**	
Metronidazole	24 (80%)
Ceftriaxone	3 (10%)
Ciprofloxacin	1 (3%)
Metronidazole and ceftriaxone	1 (3%)
Metronidazole and amoxicillin	1 (3%)

**Figure 1:**
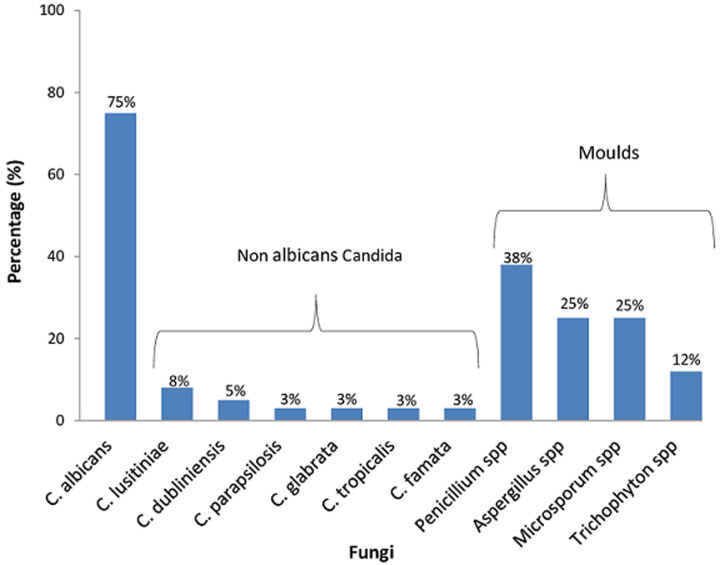
distribution of fungi isolated from diabetic foot ulcers

**Isolation of Candida, other fungi and bacteria:** out of the 152 samples collected from the foot ulcers, 31 had yeast cells which were confirmed by germ tube test (GTT) and further by VITEK - 2 System and MALDI-TOF MS. Of the 31 samples presenting with yeast cells, we isolated and differentiated 36 *Candida* species. *C. albicans* (75%, n=27) was the most frequently encountered species while *C. lusitaniae* (8%, n=3) was the predominant non-*albicans Candida* isolate followed by *C. dubliniensis* (5%, n=2). Other non-*albicans Candida* species identified included *C. glabrata, C. tropicalis, C. famata* and *C. parapsilosis* (each 2%, n=1). Majority of the *Candida* isolates were from male (24%, n=22), patients above 40 years (20%, n=23), acute wounds (21%, n=20) and ulcers of Wagner grade I and II (20%, n=26). However, this association was not statistically significant (p>0.05). Additionally, moulds were isolated from eight culture plates after 7-14 days aerobic incubation at 19-25°C. Based on colonial morphology on SDA and Lactophenol Cotton Blue (LPCB) staining technique, the moulds were identified and differentiated as *Penicillium* spp (38%, n=3), *Aspergillus* spp (25%, n=2), *Microsporum* spp (25%, n=2) and *Trichophyton mentagrophytes* (12%, n=1) (Figure 1).

Other than fungi we also identified bacteria species from 59 samples using gram staining microscopy method. Nine (15%) of these had gram positive cocci in clusters, 30 (51%) had gram negative rods while 20 (34%) of these samples had mixed bacterial infection of both gram positive cocci in clusters and gram negative rods. We further noted monomicrobial etiology (*C. albicans*) in only 4 (2.6%) of the samples compared to 27 (17.8%) of polymicrobial existence. The predominant polymicrobial etiology comprised of *C. albicans*, gram positive cocci in clusters and gram negative rods.

**Antifungal susceptibility profile:** overall, *Candida* species (n=35) were susceptible to voriconazole, flucytosine, micafungin, caspofungin, amphotericin B and fluconazole (77-97%). Among the six antifungal agents, the highest level of susceptibility was noted in flucytosine and amphotericin B while highest level of resistance was observed in caspofungin. Although the number of *C. albicans* and non-*albicans Candida* species isolated in this study did not meet the CLSI AST reporting threshold (>30 isolates each), the results were presented due to the mycological significance. *C. albicans* was resistant to caspofungin, fluconazole, micafungin and voriconazole (26%) but susceptible to amphotericin B and flucytosine (81-96%). Non-*albicans Candida* species isolated were susceptible (90-100%) to a majority of the antifungal agents tested (Table 2).

**Table 2:** antifungal susceptibility profile of *Candida* species isolated from diabetic foot ulcer patients in Kenyatta National Hospital (N=35)

Antifungal agent	Susceptibility of Candida species	
	*Candida albicans* (n=27)	Non *albicans Candida* (n=8)
Amphotericin B	81%	93%
Caspofungin	74%	90%
Flucytosine	96%	100%
Fluconazole	74%	100%
Micafungin	74%	100%
Voriconazole	74%	100%

## Discussion

The aim of this study was to determine the *Candida* species infecting DFU and assess their antifungal susceptibility pattern. *Candida albicans* was the most common species isolated while among the NAC species, *C. lusitaniae* and *C. dubliniensis* were the most prevalent. Overall, *Candida* species isolated were susceptible to voriconazole, flucytosine, micafungin, caspofungin, amphotericin B and fluconazole. The highest level of susceptibility was noted in flucytosine and amphotericin B while highest level of resistance was observed in caspofungin.

The prevalence of DFU candidiasis (20.4%) and predominance of *C. albicans* in this study is consistent with previous similar studies carried out in Turkey, India and Iran that reported prevalence ranging from 16-30% and isolated *C. albicans* as the major species [20-22]. Contrary to these findings, other studies carried out in other parts of the world reported *C. parapsilosis* as the predominant yeast in DFUs suggestive of emergence of NAC as significant pathogens [14,15]. The high frequency of *Candida* species in DFU may be attributed to immunological imbalances and increased glucose concentration in tissues and body fluids that predispose diabetic patients to fungal infections. In addition, immunomodulating action of antibiotics may support yeast survival and replication [23-25]. While we identified *C. lusitaniae* and *C. dubliniensis* as the most prevalent NAC species, these have not been previously reported in DFU [14,15].

The diversity of etiology seen in this study comprising mostly of a mixture of *Candida* species, gram negative rods, gram positive cocci and moulds may contribute to the chronic state of the wounds. This findings concur with previous studies in other parts of the world that have shown nearly all cases of DFU infections are polymicrobial [26-28]. Although the polymicrobial nature of DFU infection is not clear, it may be related to impaired immune system, gene regulation in biofilm formation and the non-fastidious nature of most of the organisms [25,26,29]. Earlier reports from India [8] and China [30] have reported monomicrobial infections comprising mostly of gram negative bacteria among acute DFU patients [14].

The emergence of multidrug and pandrug-resistant *C. albicans* and NAC noted in several parts of the world necessitates continuous antifungal susceptibility testing and monitoring. The NAC isolates in this study were 100% susceptible to voriconazole, micafungin, fluconazole and flucytosine. *C. albicans* showed high rate of resistance to the antifungals tested than NAC, similar to observations noted in earlier studies in Europe, India, and Kenya that reported a comparable resistance rate (20-48%) of *C. albicans* to triazoles [16,31,32]. Additionally, resistance to both triazole (fluconazole and voriconazole) and echinocandins (caspofungin and micafungin) group of antifungals was relatively high compared to amphotericin B and flucytosine. This observation is contrary to what has been documented in Saudi Arabia, Tunisia, and South Africa, where susceptibility rates of 96-100% to triazoles and echinocandins to both *C. albicans* and NAC species were recorded [33-35]. The differences in resistance rates noted in *C. albicans* and NAC species to triazole and echinocandins could be explained by the high clinical usage especially the fluconazole in immunocompromised patients as prophylactic drug, molecular activation of the efflux pump and mutation of *ERG11* and *ERG3* genes involved in azole target binding and accumulation of the toxic sterol 14-α-methyl-3, 6 diol [36]. Resistance in echinocandins may be attributed to mutations of *FK1* and *FK2* genes encoding for the enzyme glucan synthetase [37].

*Candida albicans* and NAC species isolated in this study showed low rates of resistance to amphotericin B similar to results of studies done in India and South Africa that reported resistance rate of 4-10% to amphotericin B [14,34]. The low incidence of resistance to amphotericin B in our study and previous similar studies could be explained by the fact that the agent is not commonly used among diabetic patients due to hypokalemia-associated nephrotoxicity. Our findings contrasts with those from other studies done in different parts of the world that reported up to 100% susceptibility rate of amphotericin B to *Candida* species [38-41]. Resistance to polyenes in *Candida* species may be associated with defective C5, 6-desaturase functionality in *C. albicans* and mutation of *ERG 2, 3, 5, 6* and *11* genes involved in ergosterol cell membrane synthesis. Different studies have shown *C. lusitaniae* to be intrinsically resistant to amphotericin B which could also explain the resistance noted among the NAC species in our study [36,42].

Generally, *Candida* species exhibit susceptibility activity to flucytosine which is used in combination with other antifungal agents in treatment of yeast infections. Resistance to this drug is slowly emerging as demonstrated in this study where *C. albicans* and NAC species showed low resistance and high susceptibility rates, respectively, to flucytosine. These findings are comparable to the low resistance rate (4-10%) and high susceptibility to flucytosine in both *C. albicans* and NAC isolates documented in studies from different tertiary hospitals in Europe, India, Iran and South Africa [14,34,43,44]. The low resistance to flucytosine observed in these studies may be due to the synergistic combination of the drug with other antifungal agents for clinical use. Resistance noted in monotherapy as shown in similar studies may be attributed to mutations of *FCY1, 2* and *FUR1* genes associated with actively transportation of the drug into the fungal cell and enzymatic conversion of the drug into 5-fluorouracil or 5-fluorouridine monophosphate [45]. It is also worth noting that the overall resistance noted to the different classes of antifungal agents in *Candida* species may also be due to biofilms formed by the organisms present in these wounds [46,47].

The main limitation in our study was the small sample size, lack of clinical information, particularly HBA_1_C to correlate with *Candida* infection, and the VITEK-2 AST panel of antifungal agents which excluded other agents. Identification of bacteria to genus and species level would have further supported our findings on polymicrobial infections; however, the scope of this study was to highlight fungal infecting agents. We would also have wished to detect the genes coding for resistance to support the resistance pattern observed.

## Conclusion

This study shows both *C. albicans* and NAC species are important etiological agents infecting diabetic foot ulcers. The study therefore provides evidence for the need to include fungal diagnosis including species identification in the investigation of diabetic foot ulcer infection. Additionally, the low level of resistance to antifungal drugs reported in this study should not be ignored because they can gradually progress to high levels, hence continuous antifungal resistance surveillance and strengthening of antifungal stewardship programmes is imperative to enhance patient care and management. In future, further research should use advanced molecular approaches to explore the diverse group of microbes infecting DFU and establish their clinical implications.

**Funding:** VMM received partial funding from Kenyatta National Hospital Research and Programs Department (Award reference number: KNH/R&P/23H/99/13) to conduct this study. The funding program or funders had no role in study design, data collection, analysis and decision in preparation and publishing this manuscript.

### What is known about this topic

- *Diabetic foot infection is polymicrobial in nature increasing the risk of lower limb amputation;*
- *Antifungal resistance, especially Candida species to azole antifungal agents is an emerging healthcare concern*.

### What this study adds

- *Candida albicans was the most frequent species isolated and showed low resistance rates to the commonly used antifungal agents;*
- *Non-albicans Candida species isolated were highly susceptible to antifungal agents tested;*
- *Isolation of yeast and moulds as fungal etiological agents infecting diabetic foot ulcers highlight the significance of including fungal diagnosis in microbiological analysis of diabetic foot infection*.
